# A Web-Based Prediction Model for Cancer-Specific Survival of Elderly Patients With Hypopharyngeal Squamous Cell Carcinomas: A Population-Based Study

**DOI:** 10.3389/fpubh.2021.815631

**Published:** 2022-01-13

**Authors:** JinKui Wang, XiaoZhu Liu, Jie Tang, Qingquan Zhang, Yuanyang Zhao

**Affiliations:** ^1^Chongqing Key Laboratory of Pediatrics, Department of Urology, Ministry of Education Key Laboratory of Child Development and Disorders, National Clinical Research Center for Child Health and Disorders (Chongqing), China International Science and Technology Cooperation Base of Child Development and Critical Disorders, Children's Hospital of Chongqing Medical University, Chongqing, China; ^2^Department of Cardiology, The Second Affiliated Hospital of Chongqing Medical University, Chongqing, China; ^3^Department of Epidemiology, Shenyang Medical College, Public Health School, Shenyang, China; ^4^Department of Otorhinolaryngology and Head and Neck Surgery, Yuhuangding Hospital of Qingdao University, Yantai, China; ^5^Department of Otolaryngology, Armed Police Hospital of Chongqing, Chongqing, China

**Keywords:** nomogram, HPSCC, elderly patients, cancer-specific survival, online application

## Abstract

**Background:** Hypopharyngeal squamous cell carcinomas (HPSCC) is one of the causes of death in elderly patients, an accurate prediction of survival can effectively improve the prognosis of patients. However, there is no accurate assessment of the survival prognosis of elderly patients with HPSCC. The purpose of this study is to establish a nomogram to predict the cancer-specific survival (CSS) of elderly patients with HPSCC.

**Methods:** The clinicopathological data of all patients from 2004 to 2018 were downloaded from the SEER database. These patients were randomly divided into a training set (70%) and a validation set (30%). The univariate and multivariate Cox regression analysis confirmed independent risk factors for the prognosis of elderly patients with HPSCC. A new nomogram was constructed to predict 1-, 3-, and 5-year CSS in elderly patients with HPSCC. Then used the consistency index (C-index), the calibration curve, and the area under the receiver operating curve (AUC) to evaluate the accuracy and discrimination of the prediction model. Decision curve analysis (DCA) was used to assess the clinical value of the model.

**Results:** A total of 3,172 patients were included in the study, and they were randomly divided into a training set (*N* = 2,219) and a validation set (*N* = 953). Univariate and multivariate analysis suggested that age, T stage, N stage, M stage, tumor size, surgery, radiotherapy, chemotherapy, and marriage were independent risk factors for patient prognosis. These nine variables are included in the nomogram to predict the CSS of patients. The C-index for the training set and validation was 0.713 (95% CI, 0.697–0.729) and 0.703 (95% CI, 0.678–0.729), respectively. The AUC results of the training and validation set indicate that this nomogram has good accuracy. The calibration curve indicates that the observed and predicted values are highly consistent. DCA indicated that the nomogram has a better clinical application value than the traditional TNM staging system.

**Conclusion:** This study identified risk factors for survival in elderly patients with HPSCC. We found that age, T stage, N stage, M stage, tumor size, surgery, radiotherapy, chemotherapy, and marriage are independent prognostic factors. A new nomogram for predicting the CSS of elderly HPSCC patients was established. This model has good clinical application value and can help patients and doctors make clinical decisions.

## Background

Hypopharyngeal carcinoma, which can occur in the pyriform sinuses bilaterally, the posterior and lateral pharyngeal walls, and the postcricoid region (1), accounts for approximately 3–5% of all head and neck carcinoma (2, 3). According to the Surveillance, Epidemiology, and End Results database (SEER), from 1973 to 2008, elderly over 65 years old with head and neck cancer accounted for about 47% of the total head and neck cancer in the United States (4). Most hypopharyngeal cancers are squamous cell carcinomas (HPSCC), which are less common than other head and neck malignancies and account for about 3–5% of squamous cell carcinomas of the head and neck. The incidence rate of cancer in the elderly increases with age and is higher in people over 65 (5, 6). So as aging intensifies, the number of elderly with HPSCC is expected to rise in the future. The prognosis of HPSCC is poor, with a 5-year survival rate being 25–47% (7, 8). Elderly patients are a heterogeneous population with their performance status (9). Comorbidity, organ dysfunction and immunosenescence as adverse factors affect the treatment options of elderly patients and let patients experience more treatment-related toxicity and poor prognosis (4, 9).

Gang et al. found that T3-4 stage, N2-3 stage, and poor tumor differentiation were risk factors for the Development of vascular invasion in HPSCC and reduced patients' overall survival (OS) and cancer-specific survival (CSS) (10). Iuchi et al. explored the modified Glasgow prognostic score as a beneficial prognostic factor for patients with HPSCC (11). Zhongyang et al. constructed a nomogram to predict survival in HPSCC and found that age, race, TNM stage, surgery, radiotherapy, chemotherapy, and marriage were independent risk factors for patients (12). Although there are nomograms to predict the prognosis of patients with head and neck cancer and even hypopharyngeal squamous cell carcinoma, there is no study to specifically construct the nomogram for elderly patients with HPSCC to predict the CSS. As a high-risk cancer population, the elderly have particular survival and prognostic factors. Therefore, a special nomogram can improve the accuracy and practical value of the prediction model.

Artificial intelligence has been widely used in human public health. Javed et al. applied Cognitive Assessment of Smart Home Resident to detect individuals with early cognitive impairment (13). Abbas et al. used a new machine-learning algorithm, BCD-WERT, to detect breast cancer, which has better accuracy than the traditional machine learning method (14).

At present, many prediction models are applied to human health, such as software metrics by Grey Wolf Optimization proposed by Yenduri et al. (15). The traditional line chart is also a commonly used prediction model. Nomogram is a visually reliable statistical tool to calculate conveniently and present the survival probability of a certain time-point for a particular patient based on risk factors included (16–18). However, there have been few Analyses of influencing factors and survival trends for hypopharyngeal cancer within the elderly population. Accurate prediction of the survival of elderly patients with HPSCC can help doctors answer patient inquiries. It also can help doctors and patients to develop treatment and follow-up strategies. We aim to explore the risk factors for CSS in elderly patients with HPSCC and construct a nomogram for predicting CSS to help doctors and patients make treatment and follow-up decisions.

## Patients and Methods

### Data Source and Data Extraction

The clinicopathological data of all patients with HPSCC from 2004 to 2018 were downloaded from the SEER database. Including age, gender, race, year of diagnosis, marriage, tumor location, tumor size, histopathological grade, histological type, TNM stage, surgery, radiotherapy, chemotherapy, and other information. Since the information in the SEE database is public and anonymous, our study does not require ethical approval and patient informed consent. Our research methods follow the guidelines of the SEER database.

### Inclusion Criteria

(1) age ≥ 60 years; (2)primary tumor site is hypopharynx; (3) histopathological type is squamous cell carcinoma.

### Exclusion Criteria

**Figure 1:**
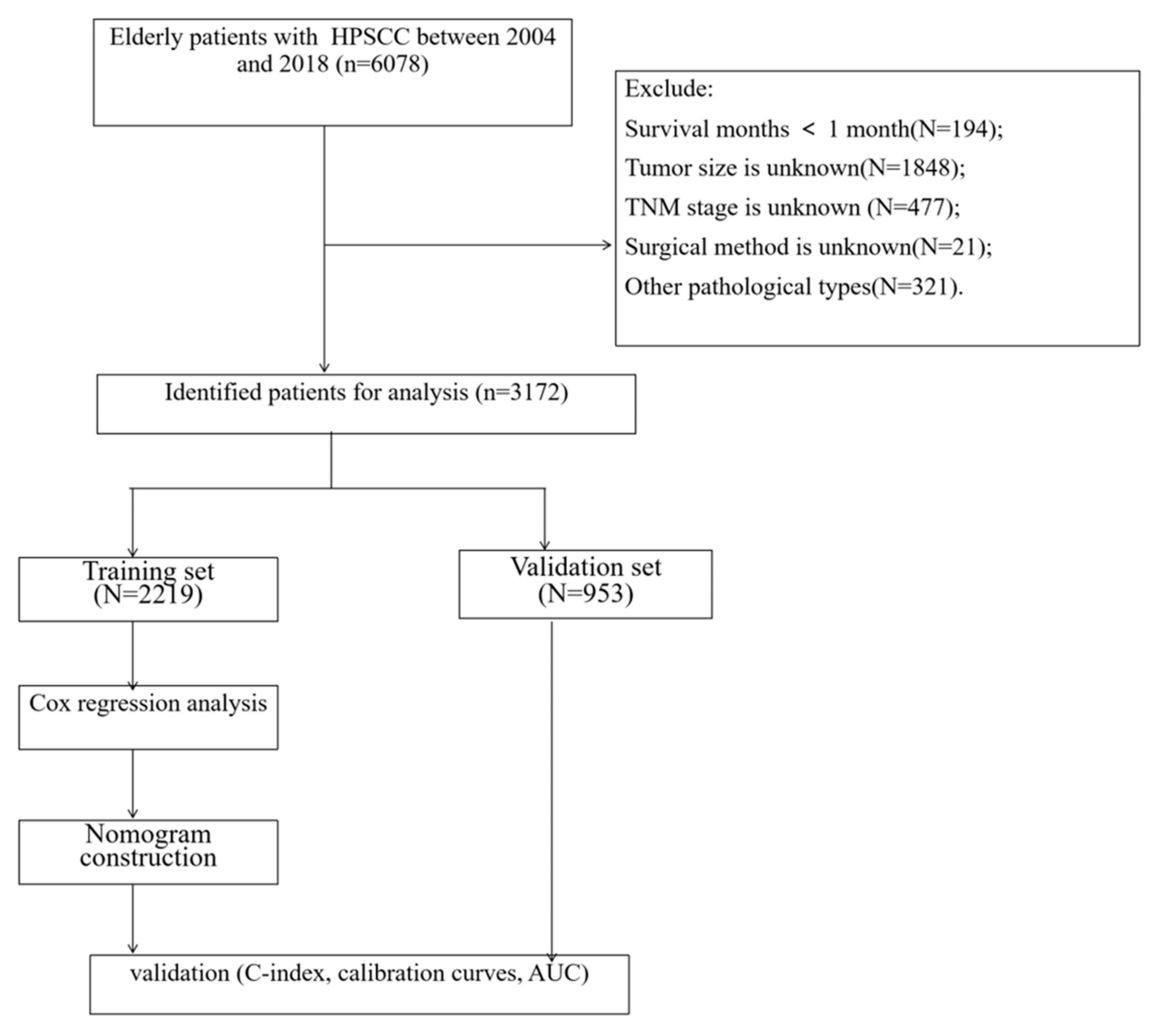
The flowchart of including and dividing patients.

(1) Survival time is <1 month; (2) tumor size is unknown; (3) TNM staging is unknown; (4) surgical method is unknown. The age of the patients was divided into three groups, including 60–69 years old, 70–79 years old, ≥80 years old. The race included white, black, and other races (American Indian/AK Indian, Asian/Pacific Islander). The tumor site was divided into the pyriform sinus and others, including the postcricoid region, aryepiglottic fold, hypopharyngeal, posterior wall of the hypopharynx. The histological grades of tumors were grade I-IV, well-differentiated, moderately differentiated, poorly differentiated, and undifferentiated. The tumor size is the most significant part of the tumor diameter, divided into ≤ 2 cm, 2–4 cm, ≥4 cm. The screening process for all patients is shown in Figure 1.

### Nomogram Construction and Validation

All patients were divided into a training set (70%) and a validation set (30%). The prognostic factors of patients need to be clarified. Cox proportional hazard regression model is the most commonly used regression model to explore the prognosis of cancer patients. The patients in the training set were subjected to univariate Cox regression analysis to screen out the prognostic factors of HPSCC. The confirmed related elements were included in the multivariate Cox regression analysis to screen out independent risk factors for HPSCC. A new nomogram was constructed to predict 1-, 3-, and 5-year CSS in elderly patients with HPSCC. Then, the consistency index (C-index) and the area under the receiver operating curve (AUC) were used to evaluate the accuracy and discrimination of the prediction model. The calibration curve of 1000 bootstrap weight samples was used to verify the accuracy of the nomogram.

### Clinical Utility

Decision curve analysis (DCA) was used to evaluate the clinical value of the nomogram. It is a new algorithm to consider the application value of the model based on various risk thresholds (19). At the same time, the clinical significance of nomogram and traditional TNM staging system was compared based on DCA. Subsequently, the patient's survival risk value was calculated based on the nomogram, and then the patients were divided into high-risk and low-risk groups based on the cut-off value. Kaplan-Meier (K-M) curve and log-rank test were used to compare the difference in survival of patients in each group.

### Statistical Analysis

All statistics were performed using SPSS 26.0 and R software 4.1.0. The count data were described by frequency (%) and analyzed by chi-square and non-parametric U-test. Univariate and multivariate Cox regression analyses were used to analyze the prognostic factors of patient survival. Kaplan-Meier curve and log-rank test were used to compare the survival differences of patients. A *P* < 0.05 was considered statistically significant.

## Results

### Clinical features

A total of 3,172 elderly patients with HPSCC were included. These patients were randomly divided into a training set (*N* = 2,219) and a validation set (*N* = 953). The clinicopathological information of the patients is shown in Table 1. Among them, 1,578 cases (49.7%) were 60–69 years old, 1,122 cases (35.4%) were 70–79 years old, and 472 cases (14.9%) were ≥80 years old. There were 2,513 whites (79.2%), 2,558 men (80.6%), and 1,590 married (50.1%). The histological tumor grades were 124 (3.91%), 1,259 (39.7%), 1,063 (33.5%), and 29 (0.91%) patients with tumor histological grades I, II, III, and IV, respectively. There were 1,635 cases (51.5%) whose tumor site was pyriform sinus. There were 747 cases (23.5%), 1,547 cases (48.8%), and 878 cases (27.7%) with tumor sizes of ≤ 2 cm, 2–4 cm, and ≥4 cm, respectively. There were 2,336 cases (73.6%) that underwent surgery, 2,393 cases (75.4%) underwent radiotherapy, and 1,863 cases (58.7%) underwent chemotherapy. There is no significant difference between the clinical-pathological information of the training set and the validation set.

**Table 1:** Clinicopathological characteristics of children with HPSCC.

	**Total**	**Training set**	**Validation set**	
	***N*** **=** **3,172**	***N*** **=** **2,219**	***N*** **=** **953**	***p***
Age				0.374
60–69	1,578 (49.7%)	1,087 (49.0%)	491 (51.5%)	
70–79	1,122 (35.4%)	801 (36.1%)	321 (33.7%)	
≥80	472 (14.9%)	331 (14.9%)	141 (14.8%)	
Race				0.536
white	2,513 (79.2%)	1,764 (79.5%)	749 (78.6%)	
black	412 (13.0%)	279 (12.6%)	133 (14.0%)	
other	247 (7.79%)	176 (7.93%)	71 (7.45%)	
Sex				0.767
Male	2,558 (80.6%)	1,793 (80.8%)	765 (80.3%)	
Female	614 (19.4%)	426 (19.2%)	188 (19.7%)	
Year of diagnosis				0.052
2004-2008	929 (29.3%)	660 (29.7%)	269 (28.2%)	
2009-2013	1,129 (35.6%)	760 (34.2%)	369 (38.7%)	
2014-2018	1,114 (35.1%)	799 (36.0%)	315 (33.1%)	
Grade				0.744
I	124 (3.91%)	88 (3.97%)	36 (3.78%)	
II	1,259 (39.7%)	869 (39.2%)	390 (40.9%)	
III	1,063 (33.5%)	749 (33.8%)	314 (32.9%)	
IV	29 (0.91%)	23 (1.04%)	6 (0.63%)	
Unknown	697 (22.0%)	490 (22.1%)	207 (21.7%)	
T				0.184
T1	408 (12.9%)	288 (13.0%)	120 (12.6%)	
T2	1,138 (35.9%)	819 (36.9%)	319 (33.5%)	
T3	619 (19.5%)	416 (18.7%)	203 (21.3%)	
T4	1,007 (31.7%)	696 (31.4%)	311 (32.6%)	
N				0.328
N0	1,117 (35.2%)	783 (35.3%)	334 (35.0%)	
N1	661 (20.8%)	451 (20.3%)	210 (22.0%)	
N2	1,278 (40.3%)	910 (41.0%)	368 (38.6%)	
N3	116 (3.66%)	75 (3.38%)	41 (4.30%)	
M				0.405
M0	2,949 (93.0%)	2,069 (93.2%)	880 (92.3%)	
M1	223 (7.03%)	150 (6.76%)	73 (7.66%)	
Marital				0.828
No	1,582 (49.9%)	1,110 (50.0%)	472 (49.5%)	
Married	1,590 (50.1%)	1,109 (50.0%)	481 (50.5%)	
Tumor size				0.041
≤ 2	747 (23.5%)	532 (24.0%)	215 (22.6%)	
2–4	1,547 (48.8%)	1,102 (49.7%)	445 (46.7%)	
≥4	878 (27.7%)	585 (26.4%)	293 (30.7%)	
Primary site				0.627
Pyriform sinus	1,635 (51.5%)	1,137 (51.2%)	498 (52.3%)	
Other	1,537 (48.5%)	1,082 (48.8%)	455 (47.7%)	
Chemotherapy				0.889
No/Unknown	1,309 (41.3%)	918 (41.4%)	391 (41.0%)	
Yes	1,863 (58.7%)	1,301 (58.6%)	562 (59.0%)	
Radiation				0.442
No/Unknown	779 (24.6%)	554 (25.0%)	225 (23.6%)	
Yes	2,393 (75.4%)	1,665 (75.0%)	728 (76.4%)	
Surgery				0.208
No	2,336 (73.6%)	1,649 (74.3%)	687 (72.1%)	
Yes	836 (26.4%)	570 (25.7%)	266 (27.9%)	

### Univariate and Multivariate Cox Regression Analysis

Univariate Cox regression analysis was used to screen the prognostic factors of HPSCC in elderly patients, including age, race, T stage, N stage, M stage, surgery, radiotherapy, chemotherapy, tumor size, and marriage. Multivariate analysis found that age, T stage, N stage, M stage, surgery, radiotherapy, chemotherapy, tumor size, and marriage were independent risk factors for the prognosis of patients. The results of univariate and multivariate factors are shown in Table 2. The results showed that older patients had a worse prognosis. The higher the TNM stage, the worse prognosis. In addition, patients with larger tumors have a worse prognosis. Patients have higher survival rates after surgery, radiotherapy, and chemotherapy. Marital status is a favorable factor for the survival of patients.

**Table 2:** Univariate and multivariate analyses of CSS in training set.

	**Univariate**			**Multivariate**		
	**HR**	**95%CI**	**P**	**HR**	**95%CI**	**P**
Age						
60–69						
70–79	1.032	0.905–1.177	0.634	1.109	0.97–1.269	0.13
≥80	1.716	1.456–2.023	<0.001	1.679	1.414–1.994	<0.001
Sex						
Male						
Female	0.93	0.8–1.08	0.361			
Year of diagnosis						
2004–2008						
2009–2013	1.001	0.871–1.149	0.991			
2014–2018	0.895	0.767–1.044	0.159			
Race						
white						
black	1.404	1.182–1.668	<0.001			
other	1.113	0.893–1.387	0.342			
Primary site						
Pyriform sinus						
Other	1.1	0.98–1.24	0.105			
Grade						
I						
II	0.913	0.68–1.227	0.546			
III	0.91	0.676–1.225	0.534			
IV	0.681	0.354–1.31	0.249			
Unknown	0.915	0.672–1.246	0.573			
T						
T1						
T2	1.328	1.065–1.655	0.012	1.264	0.965–1.657	0.089
T3	2.118	1.679–2.674	<0.001	1.635	1.204–2.221	0.002
T4	2.669	2.153–3.309	<0.001	2.196	1.644–2.935	<0.001
N						
N0						
N1	1.059	0.891–1.259	0.514	1.166	0.975–1.394	0.093
N2	1.492	1.302–1.709	<0.001	1.574	1.36–1.821	<0.001
N3	2.647	1.955–3.584	<0.001	2.296	1.68–3.137	<0.001
M						
M0						
M1	3.11	2.56–3.78	<0.001	1.992	1.622–2.447	<0.001
Surgery						
No						
Yes	0.67	0.58–0.77	<0.001	0.508	0.434–0.594	<0.001
Radiation						
No/Unknown						
Yes	0.54	0.47–0.61	<0.001	0.513	0.441–0.597	<0.001
Chemotherapy						
No/Unknown						
Yes	0.8	0.71–0.91	<0.001	0.705	0.609–0.815	<0.001
Tumor size						
≤ 2						
2–4	1.357	1.16–1.587	<0.001	1.028	0.84–1.259	0.786
≥4	2.236	1.891–2.644	<0.001	1.436	1.136–1.815	0.002
Marital						
No						
Married	0.66	0.59–0.75	<0.001	0.726	0.644–0.819	<0.001

### Nomogram Construction and Validation

**Figure 2:**
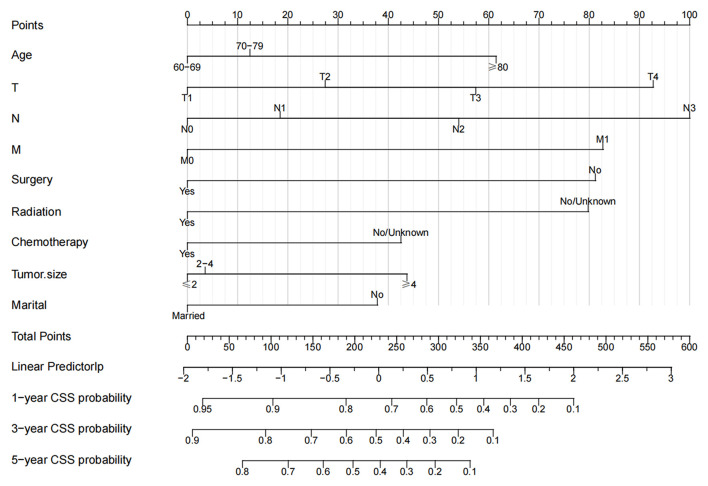
Nomogram for 1-, 3-, and 5-year CSS of elderly patients with HPSCC.

**Figure 3:**
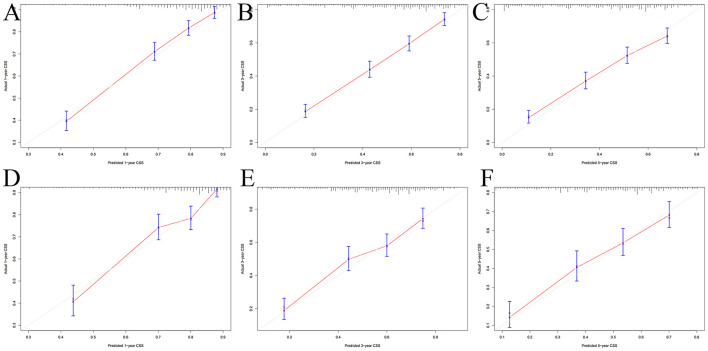
Calibration curves of the nomogram. **(A–C)** For 1-, 3-, and 5-year CSS in the training set; **(D–F)** For 1-, 3-, and 5-year CSS in the validation set.

**Figure 4:**
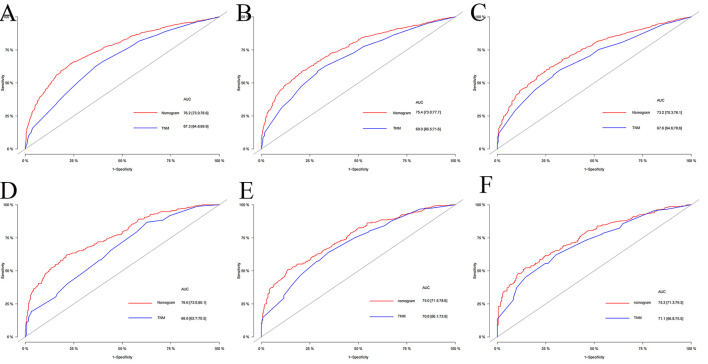
The AUC of nomogram and TNM stage of 1-, 3- and 5-year in the training set **(A–C)** and validation set **(D–F)**.

The multivariate Cox regression analysis identified the independent risk factors used to construct a nomogram to predict elderly swallowing at 1-, 3-, and 5-year CSS (Figure 2). The nomogram indicated that TNM staging is still the most significant factor affecting the prognosis of patients. In addition, surgery and radiotherapy are the most important prognostic factors among other variables. They are then followed by age, tumor size, chemotherapy, and marriage. The C-index of the training set and the validation set were 0.713 (95% CI, 0.697–0.729) and 0.703 (95% CI, 0.678–0.729), respectively, indicating that the nomogram has good accuracy. The calibration curves of the training and validation sets are consistent with the 45° diagonal, meaning that the observed value is consistent with the predicted value (Figure 3). The 1-, 3-, 5-year AUC of the training set and validation set indicates that the nomogram has better accuracy than TNM staging (Figure 4).

### Clinical Application of the Nomogram

**Figure 5:**
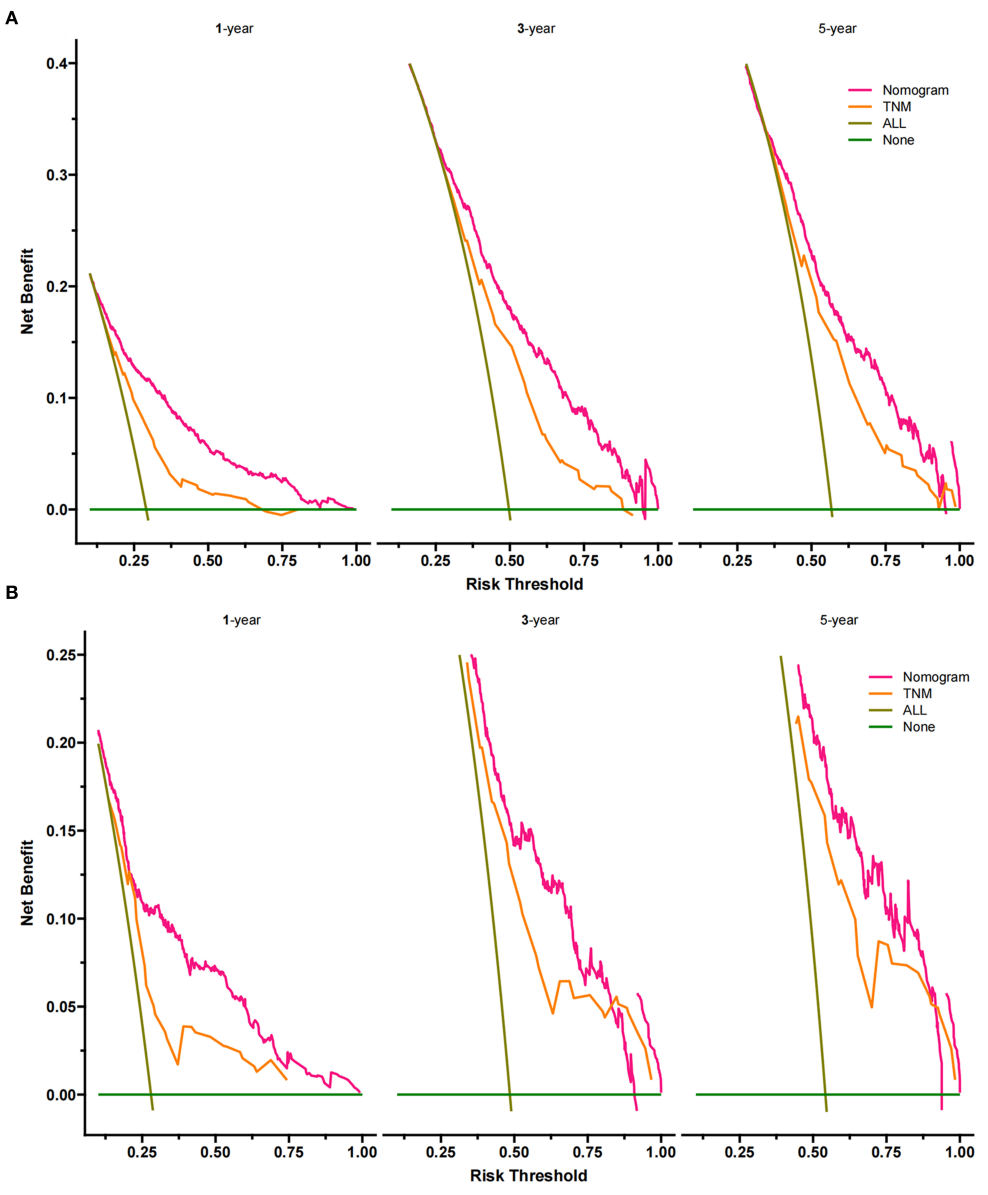
Decision curves of the nomogram predicting CSS in the training set **(A)** and validation set **(B)**. The Y-axis represents net income, and the X-axis represents threshold probability. The green line means no patients died, and the dark green line means all patients died. When the threshold probability is between 25 and 90%, the net benefit of the model exceeds all deaths or none.

**Figure 6:**
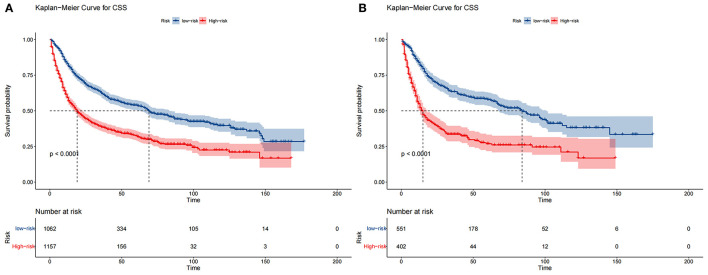
Kaplan–Meier curves of CSS for patients in the low- and high-risk groups in the training set **(A)** and validation set **(B)**.

**Figure 7:**
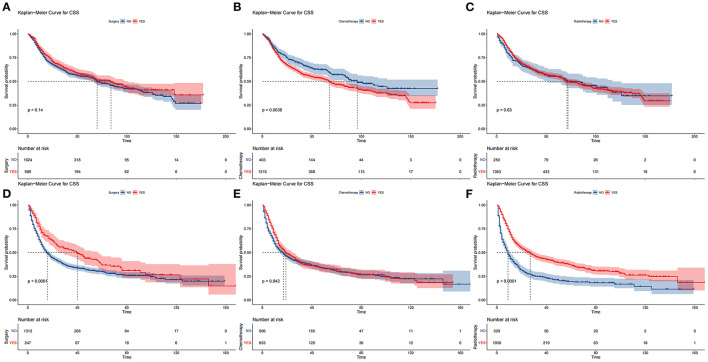
Comparison of different surgical methods of Kaplan–Meier curves. Patients with or without surgery, chemotherapy, and radiation in the low-risk group **(A–C)** and high-risk group **(D–F)**.

The DCA of the training set and the validation set still suggested that the nomogram has a better clinical application value than the traditional TNM staging (Figure 5). According to the constructed nomogram, each patient received a risk score. According to the score, patients were divided into high-risk groups (total score ≥198.9) and low-risk groups (total score ≤ 198.9). The K-M curves of the validation set and the training set proved that the survival of patients in each risk group is significantly different (Figure 6). The K-M curve result proved the discriminative power of the nomogram prediction model. We analyzed the difference in survival of patients in other risk groups under different treatments. In the low-risk group, we found no significant difference in survival between patients with and without surgery (Figure 7A). Similarly, there was no difference in survival between patients who have been treated with radiation and those who have not (Figure 7C). However, patients in the low-risk group who received chemotherapy had a lower survival rate than those who did not (Figure 7B). In the high-risk group, patients with surgery have a higher survival rate than patients without surgery (Figure 7D). Patients with radiation therapy had a higher survival rate than patients without radiation therapy (Figure 7F). Patients with chemotherapy had a higher survival rate than patients without chemotherapy (Figure 7E).

### Online Application for CSS Prediction

Based on this nomogram, we developed a network calculator at https://prediction-app.shinyapps.io/DynNomapp/. Enter the clinicopathological characteristics of the patient on this website to get the patient's predictive CSS. This website is straightforward and practical, convenient for patients and patients.

## Discussion

HPSCC is one of the most malignant head and neck carcinomas with a poor prognosis (1, 20). More than 50% of patients with HPSCC were usually diagnosed at a more advanced stage (21). Various factors lead to this result, such as minor symptoms in the early stages, early nodal metastasis, and poor differentiation (22, 23). The prognosis of HPSCC is one of the worst for head and neck tumors. The reason for this is that the hypopharynx has a rich lymphatic network, and cancer cells can metastasize through the lymphatic system at a very early stage of the disease (24, 25). Also, early diagnosis of HPSCC is often tricky as there are almost no specific clinical symptoms in the early stages. As a result, most patients are already in the advanced stages of cancer by the time the disease is diagnosed (26, 27). Elderly patients with HPSCC are a high-risk group and should receive special attention. An accurate predictive assessment can provide advice to patients and provide a basis for individualized treatment.

TNM staging is the primary tumor staging system, which is an essential factor in evaluating cancer prognosis (28). However, precise evaluation of TNM staging can only be obtained after surgery (29), and prognostic heterogeneity generally exists among patients with the same TNM stage (30). Hypopharynx is rich in lymphoid tissue and prone to mucosal infiltrative metastases (31). Sometimes, physical examination of lymph nodes is inaccurate, and small metastases cannot be detected by imaging (32), limiting prognostic evaluation accuracy by TNM staging. It should be noted that the prognosis of cancer is affected by many factors, especially in the elderly. Previous studies showed that demographics, clinicopathological parameters, and treatment factors determine survival outcomes for hypopharyngeal carcinoma (33). In our research, the multivariable Cox regression analysis revealed that age at diagnosis, marital status, tumor size, TNM stage, surgery, radiotherapy, and chemotherapy is associated with the prognosis of HPSCC.

This study found that age was still a prognostic factor for patients and that survival was lower in patients of higher age. Age as a prognostic factor is consistent with previous findings (34, 35). It is worth noting that married patients have a higher survival rate than those who are not married. Not surprisingly, marriage gives patients more emotional comfort and better financial support (36). In addition, various treatments have been shown to impact the survival of elderly patients with HPSCC significantly. Currently, radical laryngectomy is gradually becoming laryngeal preservation surgery as the procedure improves (37). Our study found that patients who had surgery still had a higher survival rate than those who did not have surgery. In addition, radiotherapy and chemotherapy offer patients the option of laryngeal preservation surgery (38). Our study showed that patients had significantly better survival rates with radiotherapy and chemotherapy.

In addition, patients were divided into high-risk and low-risk groups based on the scores of the line graphs. We found that surgery and radiotherapy for patients in the low-risk group did not significantly improve patient survival. However, chemotherapy appeared to improve the survival prognosis of elderly patients significantly. Therefore, chemotherapy should be routinely recommended for elderly patients with HPSCC in the low-risk group. For patients in the high-risk group, surgery, radiotherapy, and chemotherapy are all necessary as they effectively improve patient survival.

Although nomograms have been constructed to predict OS and CSS in hypopharyngeal cancer, no nomograms have been explicitly built to expect CSS in elderly patients with HPSCC (39, 40). Due to the population-specific nature of the elderly, there may be prognostic factors that differ from those of other populations. Therefore, based on these factors, we developed a nomogram to predict the prognosis of the elderly with HPSCC. The calibration curve and C-index of the prediction model suggest good accuracy and reliability. Based on the AUC and DCA results, this new nomogram is superior to conventional TNM staging.

Our research also has some defects. First, there is no information about the comorbidity of the elderly in the SEER database, such as coronary heart disease, hypertension, and diabetes. These complications affect the choice of treatment options. We cannot incorporate these factors into the prognosis analysis, so the accuracy is limited. However, we included essential patient factors such as age, TNM stage, surgery, radiotherapy, chemotherapy, etc. These factors are critical determinants of patient survival. Second, as this study was a retrospective case study, a selection bias was difficult to adjust for and may have contributed to some errors in the results. Finally, although we have conducted split internal validation on the SEER database, we lack multi-center external validation, especially data validation from China. This limits the application of the nomogram in areas other than the United States. We intend to further validate the accuracy of the prediction model through external patient data. The data were collected prospectively according to the variables of this model, and the patients were further followed up. Then the patients were included in the model for external validation.

## Conclusion

In conclusion, we established and validated a new nomogram to predict the prognosis of elderly patients with HPSCC. Through this model, clinicians can estimate the survival rate of elderly patients with HPSCC more accurately, which plays an essential role in guiding the choice of clinical treatment.

## Data Availability Statement

Publicly available datasets were analyzed in this study. This data can be found here: https://seer.Cancer.gov/.

## Author Contributions

JW, YZ, and XL contributed to the conception and design. JW, JT, and XL collected and analyzed the data. JW, QZ, and JT drew the figures and tables. JT, YZ, and JW wrote the draft. XL and JW contributed to manuscript writing and revision. All authors approved the final manuscript.

## Conflict of Interest

The authors declare that the research was conducted in the absence of any commercial or financial relationships that could be construed as a potential conflict of interest.

## Publisher's Note

All claims expressed in this article are solely those of the authors and do not necessarily represent those of their affiliated organizations, or those of the publisher, the editors and the reviewers. Any product that may be evaluated in this article, or claim that may be made by its manufacturer, is not guaranteed or endorsed by the publisher.
